# Novel Synthetic DNA Immunogens Targeting Latent Expressed Antigens of Epstein–Barr Virus Elicit Potent Cellular Responses and Inhibit Tumor Growth

**DOI:** 10.3390/vaccines7020044

**Published:** 2019-05-24

**Authors:** Krzysztof Wojtak, Alfredo Perales-Puchalt, David B. Weiner

**Affiliations:** 1 Vaccine and Immunotherapy Center, The Wistar Institute, Philadelphia, PA 19104, USA; kwojtak@Wistar.org (K.W.); Alfredo.PeralesPuchalt@inovio.com (A.P.-P.); 2 Cell and Molecular Biology Graduate Program, The University of Pennsylvania, Philadelphia, PA 19104, USA

**Keywords:** Epstein-Barr virus, DNA vaccines, latent proteins, LMP2, EBNA1, LMP1

## Abstract

Infectious diseases are linked to 15%–20% of cancers worldwide. Among them, Epstein–Barr virus (EBV) is an oncogenic herpesvirus that chronically infects over 90% of the adult population, with over 200,000 cases of cancer and 150,000 cancer-related deaths attributed to it yearly. Acute EBV infection can present as infectious mononucleosis, and lead to the future onset of multiple cancers, including Burkitt lymphoma, Hodgkin lymphoma, nasopharyngeal carcinoma, and gastric carcinoma. Many of these cancers express latent viral genes, including Epstein–Barr virus nuclear antigen 1 (*EBNA1*) and latent membrane proteins 1 and 2 (LMP1 and LMP2). Previous attempts to create potent immunogens against EBV have been reported but generated mixed success. We designed novel Synthetic Consensus (SynCon) DNA vaccines against EBNA1, LMP1 and LMP2 to improve on the immune potency targeting important antigens expressed in latently infected cells. These EBV tumor antigens are hypothesized to be useful targets for potential immunotherapy of EBV-driven cancers. We optimized the genetic sequences for these three antigens, studied them for expression, and examined their immune profiles in vivo. We observed that these immunogens generated unique profiles based on which antigen was delivered as the vaccine target. EBNA1vax and LMP2Avax generated the most robust T cell immunity. Interestingly, LMP1vax was a very weak immunogen, generating very low levels of CD8 T cell immunity both as a standalone vaccine and as part of a trivalent vaccine cocktail. LMP2Avax was able to drive immunity that impacted EBV-antigen-positive tumor growth. These studies suggest that engineered EBV latent protein vaccines deserve additional study as potential agents for immunotherapy of EBV-driven cancers.

## 1. Introduction

Epstein–Barr virus (EBV) is a large double-stranded DNA gammaherpesvirus with about 170 kilobases in its genome, encoding over 100 open reading frames (ORFs). EBV accounts for about 1% of all cancer cases worldwide. This complex virus is ubiquitous in the human population, establishing a lifelong latent infection in 90% of people by adulthood [1,2]. The viral strains can be divided into two subgroups, type 1 and type 2, which are broadly similar and designated by differences in their nuclear antigens [3,4]. Primary infection is either asymptomatic, experienced as a non-specific infection, or the cause of infectious mononucleosis, with the latter more likely if exposure occurs during adolescence or later [5]. EBV targets human B cells after being transmitted through the oral epithelium via the saliva of an infected individual, establishing latency and allowing the viral genome to persist.

EBV is linked to the development of several human cancers. It was first identified in a Burkitt lymphoma sample [6], and is now known to be a cause of Hodgkin’s lymphoma [7], nasopharyngeal carcinoma [8,9], and gastric carcinoma [10]. EBV infection is also linked to autoimmune disorders, such as multiple sclerosis [11] and systemic lupus erythematosus [12], which are likely tied to EBV-driven immune dysregulation [13]. The cancers associated with EBV are linked to their expression of EBV oncogenes, including Epstein–Barr virus nuclear antigen 1 (EBNA1) and latent membrane proteins 1 and 2 (LMP1 and LMP2) [14]. The latent viral oncoproteins of EBV are important cancer drivers and are implicated in directly contributing to EBV-associated malignancies [15,16,17]. 

EBNA1 is important in maintaining the viral genome and is required for EBV latency and associated transformation. LMP1 and LMP2 were discovered to colocalize in the membranes of latently infected lymphocytes [18], and these oncoproteins contribute to cancer progression via diverse signaling pathways [19]. LMP1 interacts with tumor necrosis factor receptor (TNFR)-associated factors (TRAFS) to drive nuclear factor-κB (NF-κB), mitogen-activated protein kinase (MAPK), and phosphatidylinositol 3-kinase (PI3K) pathways [20]. LMP2 mimics the B cell receptor, sending survival signals to B cells without the need for antigen stimulation [21]. The LMP2 gene produces LMP2A and LMP2B, of which LMP2A has an additional 119 amino acids at the N-terminus. 

There are no approved vaccines available to prevent initial infection by EBV, and clinical trials of EBV vaccine candidates have had limited success. The target that progressed furthest along in the clinic was a recombinant subunit gp350 prophylactic vaccine adjuvanted with aluminum hydroxide and 3-O-desacyl-4′-monophosphoryl lipid A (AS04) which was tested in a phase 2 trial. The study reported that it statistically decreased the incidence of infectious mononucleosis, but this vaccine did not reduce infections by the virus, despite generating high-titer antibody responses in vaccine recipients [22]. Future vaccines against EBV can further explore the numerous other glycoproteins involved in EBV entry and the latent proteins essential for maintaining the virus [23]. 

EBV is a viable target for therapeutic approaches to treating cancer. Cellular immune responses are particularly important in targeting malignant cells, and they have been exploited in specific cancer immunotherapies [24,25]. It would be a major advantage for such approaches if they would drive both CD4 T cell responses and induce functional CD8 T cell responses that could clear EBV-infected targets. Prior vaccine approaches particularly lacked potent induction of CD8 cellular immunity. 

Newer Synthetic Consensus (SynCon) DNA vaccines, combined with adaptive electroporation (EP), have demonstrated safety, as well as the potent induction of antibodies, T helper responses, and CD8 effector T cells, in multiple clinical trials. Clinical efficacy has been reported in the context of immunotherapy for human papillomavirus (HPV)-driven neoplasia, and clinical regressions with clearance have been described in early studies that use a combination approach involving engineered HPV nuclear gene targets and checkpoint inhibitor therapy with PD-1. Specifically, a therapeutic DNA vaccine targeting HPV E6/E7 antigens from the HPV 16 and 18 strains has shown a positive impact in patients when this vaccine was delivered by Cellectra adaptive EP in a phase 2b trial for the treatment of cervical intraepithelial neoplasia [26]. Importantly, this vaccine induced potent CD8 T cells that infiltrated the tumor and caused the lesions to regress, resulting in both histopathological regression and viral clearance in 40% of treated patients. Similar data has been reported impacting HPV-driven head and neck cancers in a preliminary report [27], where a similar genetically-adjuvanted HPV DNA vaccine has been shown to drive an increase in intratumoral T cell infiltration by CD8 cells, as well as result in complete clinical regression in metastatic head and neck cancer when the vaccine was followed by PD-1 immunotherapy (this outcome was observed in 2/4 patients). 

These data support the importance of the synthetic DNA approach for the treatment of virally-driven cancers which rely on viral oncogenes for continued disease. This is the situation for EBV-driven cancer as well. Here we report on studies investigating the generation of a multiantigen immunotherapeutic vaccine for EBV infection. We focused on developing a vaccine cocktail consisting of the episome-maintaining EBNA1 antigen combined with the two important latency-related membrane antigens for EBV, LMP1 and LMP2. We report the immune potency and early impact of the combined immune responses to these constructs. 

DNA vaccines have previously reported interesting responses against LMP1 [28] and LMP2 [29] in mouse models. This study furthers this research by exploring the immune responses to a combination of EBV latent proteins using newly designed synthetic DNA-encoded antigens studied in the context of facilitated in vivo local delivery. The results show potent and consistent induction of T cell immunity in targeted mouse models with an impact on antigen-positive tumor growth, suggesting further study of this approach for EBV immunotherapy is important.

## 2. Materials and Methods 

### 2.1. DNA Vaccines

Latent protein vaccine consensus sequences for EBNA1vax, LMP1vax, and LMP2Avax were produced from sequences obtained from strains AG876, B95-8, and GD1. Codons corresponding to residues associated with cell signaling were modified. Repetitive sequences were deleted from the EBNA1vax consensus sequence to avoid their inhibition of translation and MHC class I presentation [30,31,32], and alanine mutations were made, affecting binding to USP7 [33]. Similarly, mutations were made to functional domains of LMP1vax and LMP2Avax to avoid signaling through potentially oncogenic pathways [34,35,36,37,38,39]. The sequences were codon optimized using SynCon technology and prepared for vaccination studies within modified pVAX1 plasmids, as previously described [40]. 

### 2.2. Western Blots

Proteins were extracted, denatured, and immunoblotted as previously described [41]. Detection antibodies used were anti-LMP2A clone 15F9 (Biorad, Hercules, CA, USA), anti-LMP1 clone CS 1-4 (Abcam, Cambridge, UK) and a polyclonal anti-EBNA1 antibody (Invitrogen, Carlsbad, CA, USA). Secondary anti-rat, -mouse, and -goat antibodies conjugated to horseradish peroxidase were used for visualization. Anti-β-actin (a5441, Sigma-Aldrich, St. Louis, MO, USA) was used as a loading control. Images were captured using an ImageQuantLAS 4000 (GE Healthcare Life Sciences, Marlborough, MA, USA). 

### 2.3. Immunofluorescence

Cover slides coated in poly-L-lysine had 293T cells grow on them in 12-well plates and they were transfected with pVAX empty vector, EBNA1vax, LMP1vax, or LMP2Avax DNA vaccine plasmids using Lipofectamine 2000 per the manufacturer’s protocol (Invitrogen, Carlsbad, CA, USA). After incubating for two days, the cells were washed with phosphate-buffered saline (PBS), fixed with 4% paraformaldehyde, and permeabilized using Triton X-100 in PBS, as previously described [42]. Commercial antibodies to EBNA1, LMP1, and LMP2A were used for primary staining as above and Invitrogen anti-mouse, anti-rat, and anti-goat secondary antibodies conjugated to AF488, AF647, and APC were used. Slides were imaged using a Leica TCS SP5 Confocal Laser Scanning Microscope and analyzed with Leica LAS AF software (Leica Microsystems, Wetzlar, Germany). 

### 2.4. ELISPOT

Mouse splenocytes were incubated for 24 hours with peptide pools composed of 15mers overlapping by 11 amino acids and covering the full EBNA1, LMP1, and LMP2A proteins (PepTivator EBV, Miltenyi Biotec, Bergisch Gladbach, Germany). Peptides were resuspended at 5 μg/mL during stimulation. IFNγ ELISPOT was performed according to the manufacturer’s instructions. Spots were counted using a Cellular Technology Limited ImmunoSpot Analyzer, as previously described [43]. 

### 2.5. Flow Cytometry

Two million splenocytes were cultured for 5–6 hours with the peptide pools used above, as previously described [44], and with eBioscience protein transport inhibitor cocktail (Invitrogen). Surface (for CD4 and CD8) and intracellular (for remaining markers) staining followed. Biolegend anti-mouse antibodies conjugated to fluorophores used in this experiment included CD3ε-PE/Cy5 (145-2C11), CD4-FITC (RM4-5), CD8a-APC/Cy7 (53-6.7), IFNγ-APC (XMG1.2), TNFα-BV605 (MP6-XT22), and IL-2-PE-Cy7 (JES6-5H4). Live-dead exclusion was performed using violet fluorescent reactive dye (Invitrogen). Data was collected using a BD Biosciences LSRII flow cytometer (BD Biosciences, Franklin Lakes, NJ, USA) and analyzed using FlowJo v10 (FlowJo LLC, Ashland, OR, USA). 

### 2.6. Cell Lines

Retroviruses encoding B95-8 LMP2A and a green fluorescent protein (GFP) reporter were produced by transfecting Phoenix cells (ATCC) with LMP2A sequence in pBMN-I-GFP. The retrovirus-containing media harvested from these cells was used to infect TC-1 cells by spin-infection to generate a tumor cell line, as previously described [45], which stably expresses LMP2A. Cells expressing the GFP marker were isolated using FACS, and single-cell cloning was performed to obtain a clonal cell population. 

### 2.7. Animal Studies

Female, 5-7-week-old C57BL/6 and BALB/c mice were purchased from Jackson Labs, and CD-1 mice were purchased from Charles River. The Wistar Institute Institutional Care and Use Committee approved all animal studies under protocol 112762. 

Tumors were generated by injecting 2 million TC-1-LMP2A cells into the axillary region, with monitoring of tumor size thereafter. Tumor sizes were measured by taking their longest dimension as length and the perpendicular as width, with tumor volume being calculated using ½ × length × width^2^. Multifocal tumors were separately measured, and their total volume was calculated as the sum of the individual volumes. Vaccinations introduced 25 μg of DNA delivered within 30 μL of deionized water by intramuscular injection into the tibialis anterior and were followed by EP with the Cellectra 3P device (Inovio Pharmaceuticals) under general anesthesia using inhaled isoflurane, as previously described [46,47]. Blood was collected through submandibular bleeding or post-mortem cardiac punctures. 

### 2.8. Statistics

GraphPad Prism 7 and 8 were used to perform statistical analyses. The two-tailed unpaired Student’s t test was used to calculate differences between means of experimental groups, with the Mann–Whitney test for non-parametric distributions. One-way analysis of variance (ANOVA) was used for comparisons between more than one group, with Kruskal-Wallis used in cases of nonparametric distributions. Error bars in all graphs show the standard error of the mean. The log-rank test was used to compare survival rates. *p* < 0.05 was considered statistically significant.

## 3. Results

### 3.1. Design of DNA Vaccines Targeting EBNA1, LMP1, and LMP2A

**Figure 1:**
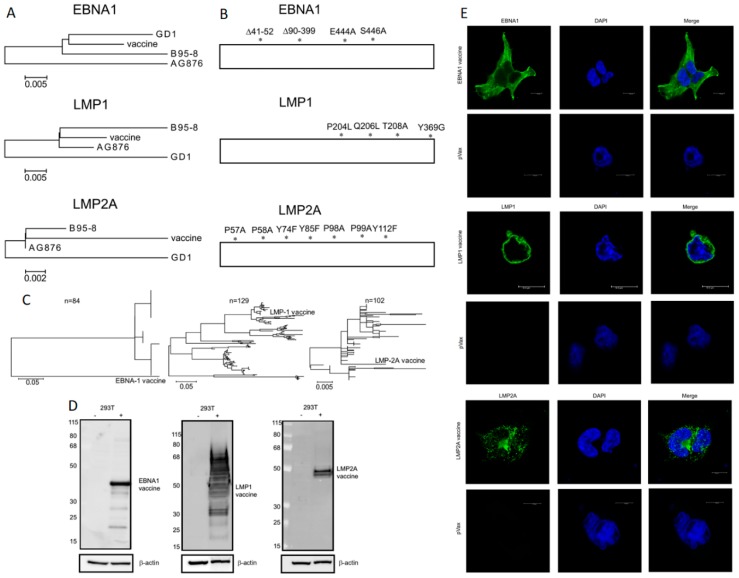
Design and expression of EBNA1vax, LMP1vax, and LMP2Avax vaccine antigens. (**A**) Diagram showing the similarity of the consensus sequence of the EBNA1, LMP1, and LMP2A vaccines, generated from the sequences of EBV strains B95-8, AG876, and GD1. The vaccine antigen designs use a SynCon sequence embedded in a pVAX plasmid. (**B**) Modifications were made to the consensus vaccine antigens to avoid potentially oncogenic properties and repetitive sequences. (**C**) Phylogenic trees showing relationship of vaccines to known EBV latent protein sequences. (**D**) Western blots showing the expression of vaccine antigens in untransfected cells (left columns) and cells transfected with the DNA vaccine (right columns). Beta-actin was used as a loading control. (**E**) Immunofluorescence images showing expression of the vaccine antigens in 293T cells, with cytoplasmic EBNA1vax, LMP1vax on the outer membrane, and LMP2Avax showing a vesicular localization. Antigens are labeled in green, and DAPI (4′,6-diamidino-2-phenylindole) shows the nucleus in blue. Scale bars are 10 μm.

We designed consensus optimized DNA vaccines targeting the oncogenic EBV latent proteins commonly seen in malignancies, which are EBNA1, LMP1, and LMP2A. Consensus immunogens can focus the immune response towards conserved regions of important antigens, allowing for increased T cell cross reactivity as well as partially compensating for minor variability in the vaccine targeted antigens [48,49,50]. Consensus sequences using *GD1* (type 1), *B95-8* (type 1), and *AG876* (type 2) EBV genes were generated for all 3 antigens (Figure 1A) to optimize the ability of the vaccines to elicit immune responses against all common viral strains, which are phylogenetically similar [51]. Modifications were made to remove repetitive sequences and to ablate oncogenic properties inherent to the proteins while preserving the structures of the antigens (Figure 1B). EBNA1vax had repetitive sequence removed, and all three antigens had amino acids modified to abrogate functional regions and cell signaling pathways (Appendix Figure A1). Phylogenetic trees show close relationships between the vaccine antigens and known sequences from viral isolates (Figure 1C). Large deletions were made to repetitive sequences when engineering EBNA1vax, leading to divergence from known EBNA1 sequences and the long branch away in the diagram, although the retained sequences are well-conserved. The LMP vaccines lie well within their phylogenetic trees, with LMP1 demonstrating roughly 10-fold more diversity than LMP2A. This conservation supports the likelihood that the targeted changes will elicit immune responses against native EBV antigens, as we have described in the clinic for HPV [26,27], Ebola [52], and Zika [53]. However, formal testing in animal models and evaluation in humans is important.

### 3.2. In Vitro Expression of DNA Vaccines

293T cells were transfected with the vaccine DNA plasmids to test for expression of the designed synthetic DNA constructs. Western blots of lysates from the transfected cells showed bands for EBNA1vax, LMP1vax and LMP2Avax vaccines close to their predicted molecular weights (Figure 1D). We performed immunofluorescence on the transfected 293T cells to further evaluate the expression and localization of the constructs. These studies confirmed expression of all 3 proteins, with LMP2Avax showing its characteristic granular distribution and LMP1vax displaying membrane expression (Figure 1E). Interestingly, EBNA1vax was found in the cytoplasm instead of with the typical nuclear localization of EBNA1. This difference may be due to the changes to the consensus sequence aimed at avoiding sequence repeats and specific changes in the functional domains that affect the ability of EBNA1vax to bind to DNA, suggesting that the encoded changes result in attenuation.

### 3.3. Inbred Mice Produced Significant Responses to Latent Protein DNA Vaccines

**Figure 2:**
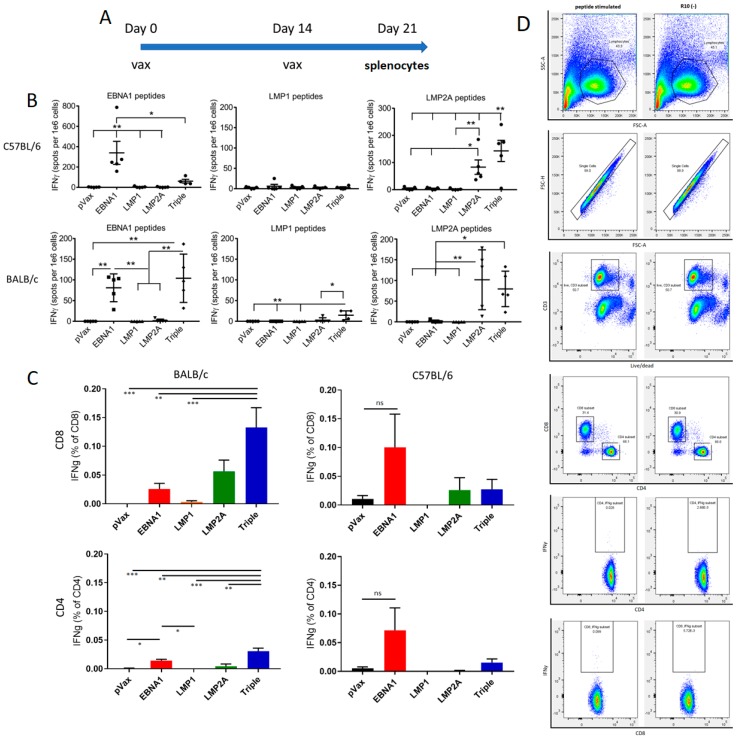
DNA vaccination produces strong cellular responses in inbred mice. (**A**) Vaccination schedule to test the immunogenicity of latent proteins in inbred mice. 2 doses of individual or combined latent protein vaccines (vax) were given to groups of 5 BALB/c or C57BL/6 mice two weeks apart, with mouse splenocytes being harvested one week after the final dose (sac). (**B**) Cellular responses of BALB/c and C57BL/6 mice measured using IFNγ ELISPOT after overnight stimulation with peptide pools. Responses were minimal for LMP1vax, but much larger for EBNA1vax and LMP2Avax. (**C**) Cellular response measured by flow cytometry. IFNγ staining of cells was measured following their stimulation with latent protein peptides. Pooled EBNA1, LMP1, and LMP2A peptides were used for stimulation. (**D**) The gating of representative examples of the BALB/c CD8 data is shown. Peptide stimulated splenocytes from a mouse vaccinated with the combination vaccine are shown on the left, and control cells left in media are shown on the right. *p < 0.05, **p < 0.01, ns: not significant.

In vivo immune responses to EBNA1vax, LMP1vax, and LMP2Avax were examined in BALB/c and C57BL/6 mice. The animals were vaccinated with either the empty vector, individual EBNA1vax, LMP1vax, or LMP2Avax vaccine antigens, or a combination vaccine incorporating all three plasmids. Groups of five mice received biweekly vaccinations, and a week after the second dose they were sacrificed to have their splenocytes collected for analysis (Figure 2A). 

IFNγ responses to latent protein peptide pools were evaluated using an ELISPOT assay (Figure 2B). Splenocytes from mice vaccinated with EBNA1vax generated an average of 81 spot forming units (sfu) per million cells for the individual vaccine and 104 sfu for the combined triple vaccine in BALB/c mice, an insignificant difference. A more robust 340 sfu were observed for the same vaccine in C57BL/6 mice, whereas the combination vaccine was much less immunogenic, suggesting that other antigens in the mixture were more a focus of the immune response. LMP2Avax generated responses in both mouse strains as an individual vaccination and in combination with the other antigens. BALB/c mice showed 102 sfu for the individual vaccine and 80 sfu for the combined, and C57BL/6 mice exhibited 83 sfu for LMP2A vax alone and 178 sfu in combination. LMP1vax produced a more modest response of 15 sfu in BALB/c mice that was only notable in the combination vaccine and not observed in the C57BL/6 animals. The modifications that were made to LMP1vax may have limited its immunogenicity. Additional engineering was undertaken to enhance the immunity of the LMP1 antigen. Modified constructs involved the inclusion of an IgE leader sequence coincident with truncation of the N-terminal native sequence, as well as inclusion by gene fusion of tetanus toxoid fragments as part of the ORF. Two constructs were made, one with a short peptide fragment inserted at the C-terminus (LMP1tt30) and the other with a 256 amino acid fragment inserted after the leader sequence (LMP1ttDOM). This design improved the immunity generated by the fusion antigen vaccine (Appendix Figure A2). 

Evaluation of IFNγ by flow cytometry was showed that CD8 cells were driving the immune response (Figure 2C). The triple vaccine generated more robust CD4 and CD8 responses in BALB/c mice, with greater CD8 responses than in the C57BL/6 mice. Overall, the responses induced appeared to be more potent for the induction of CD8 T cell immunity, with a smaller percentage of CD4 T cell induction, suggesting the vaccine is CD8 T cell biased. Gating for the flow cytometry data is shown in Figure 2D.

### 3.4. CD8 Cellular Responses Were Robust in Outbred CD-1 Mice

**Figure 3:**
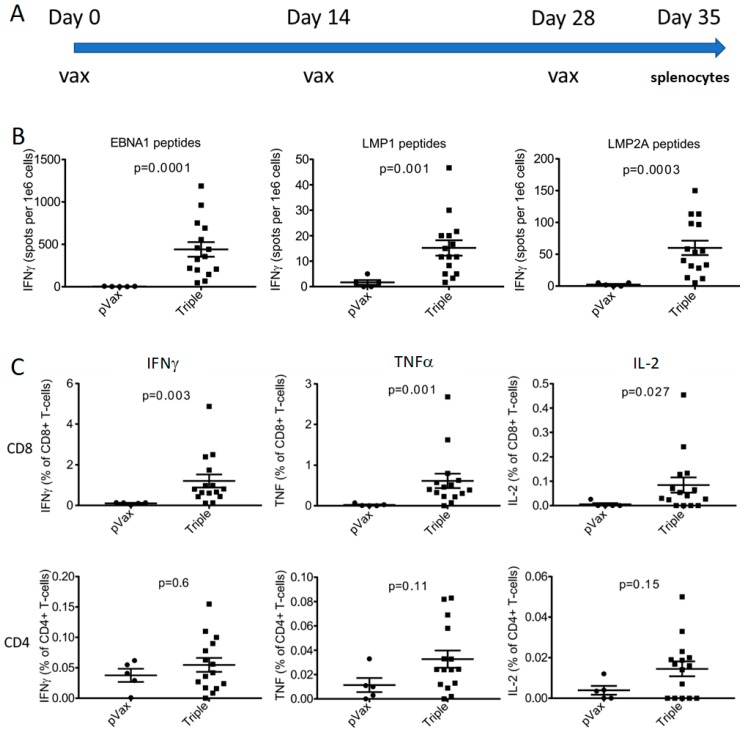
Cellular responses produced by combination vaccine in outbred CD-1 mice. (**A**) Vaccination schedule in outbred CD-1 mice. Mice were vaccinated with a combination of EBNA1vax, LMP1vax, and LMP2Avax three times at biweekly intervals, followed by harvesting of their splenocytes a week after the final vaccination. (**B**) Cellular responses to respective peptide pools, shown by IFNγ ELISPOT. (**C**) Plots showing CD4 or CD8 responses of CD-1 mice immunized with the triple vaccine or empty vector (pVax), stimulated with pooled peptides derived from EBNA1, LMP2A and LMP1. Cellular responses are driven by CD8+ cells, as shown by flow cytometry following stimulation of splenocytes.

To further study these immunogens in a more relevant outbred animal model, we next vaccinated CD-1 mice and compared their responses to control-vaccinated animals. These mice were vaccinated three times at two-week intervals, and immune studies were performed a week after the final vaccination (Figure 3A). Cellular responses were once again more robust for EBNA1 and LMP2A than for LMP1, as was again observed in the inbred mouse models. However, stimulation with each of the latent protein peptide pools produced some responses, as measured by IFNγ ELISPOT (Figure 3B). CD8 responses were dominant when the splenocytes were analyzed by flow cytometry, and CD4 responses were lower (Figure 3C). The CD-1 response supports the CD8 potency of this vaccine approach.

### 3.5. LMP2Avax Delays Tumor Growth

**Figure 4:**
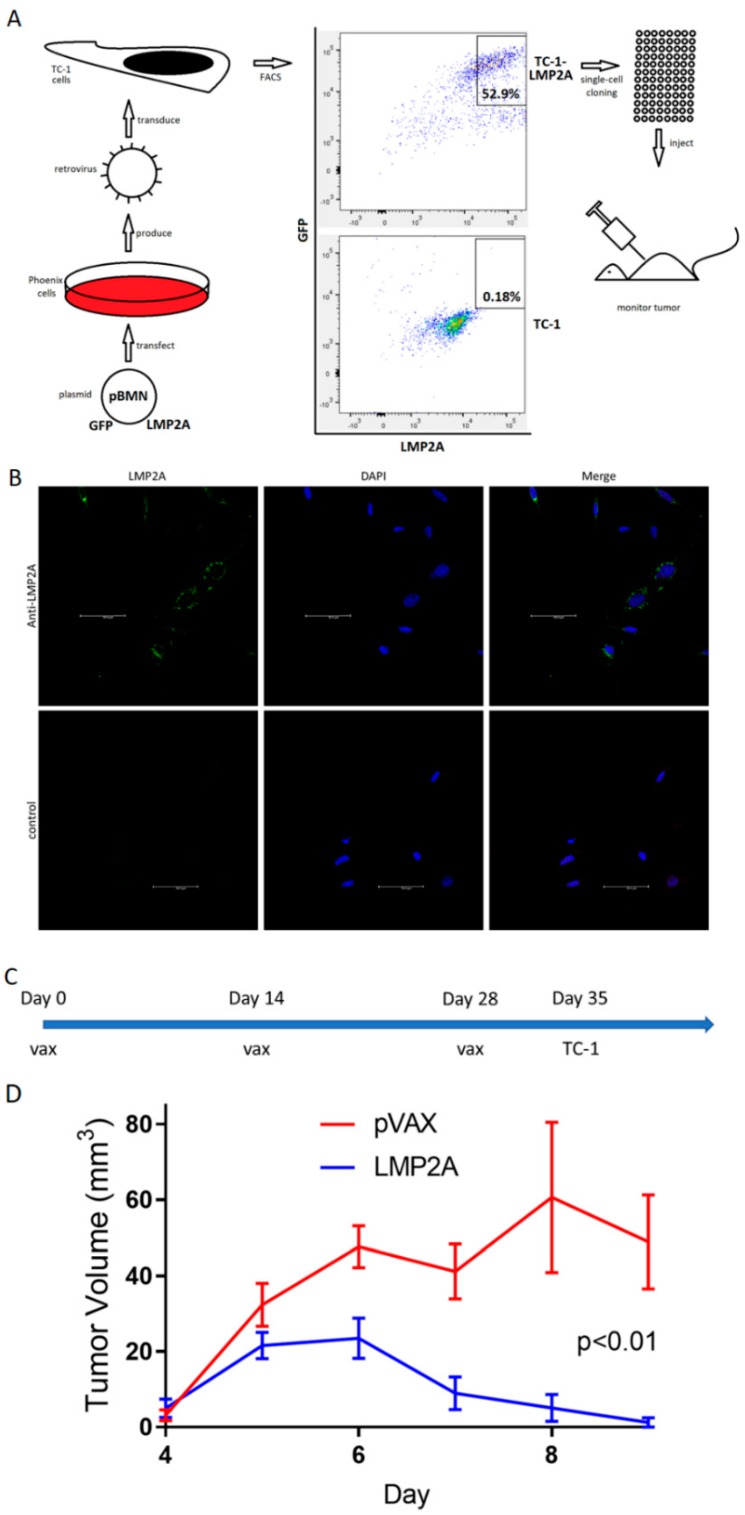
LMP2Avax inhibits tumor growth in mice. (**A**) Workflow to produce tumor cell lines expressing target antigen, in this case TC-1-LMP2A. (**B**) Immunofluorescence assay demonstrating LMP2A expression in TC-1-LMP2A cell line. DAPI is shown in blue, with LMP2A labeled in green. Anti-LMP2A antibodies were used as primary Abs (top), with secondary Abs conjugated to AF647. Anti-EBNA1 primary antibodies were used as a negative control (bottom). Scale bars are 50 μm. (**C**) Vaccination schedule prior to tumor introduction. Two groups of five C57BL/6 mice received three biweekly vaccinations followed by the subcutaneous axillary injection of 2 million TC-1 cells stably expressing LMP2A. The vaccines used 20 μg of DNA in 30 μL of water delivered by electroporation, with the vaccine group receiving plasmid encoding LMP2Avax and the control receiving the empty vector pVAX. Tumor sizes were monitored daily afterwards. (**D**) TC-1-LMP2A tumor volume over time in mice vaccinated with LMP2A or empty vector. Bars show scanning electron microscopy (SEM).

In order to study the possible impact on an EBV+ tumor expressing a model LMP2A antigen, we next generated a murine epithelial tumor cell line using TC-1 cells that were constructed to express LMP2A using a retroviral transduction system. This may cause high expression of LMP2A relative to EBV-associated tumor cells, but LMP2A protein is expressed in the cancer cells of patients and its epitopes are recognized by T cells [54]. This cell line serves as a vaccine target for our LMP2A immunogens. We generated and selected the LMP2A line as shown in Figure 4A. Briefly, retroviral vectors produced by transfecting Phoenix cells with pBMN plasmids containing GFP and LMP2A were used to stably transduce TC-1 cells. These cells underwent selection via fluorescence-activated cell sorting (FACS) and single-cell cloning to produce a homogenous population expressing LMP2A, and this population was used to introduce tumors into mice. 

The expression of LMP2A in the derived TC-1-LMP2A tumor line was confirmed by antibody reactivity as demonstrated by immunofluorescence in Figure 4B. We next studied the use of this cell line as a tumor challenge antigen. C57BL/6 mice received either the LMP2Avax DNA vaccine or empty vector three times at biweekly intervals, followed by an axillary injection of 2 million tumor cells after the final vaccination (Figure 4C). LMP2Avax vaccinated mice showed a smaller tumor volume and more rapid tumor shrinkage than those vaccinated with the empty vector, demonstrating the anti-tumor immunogenic potential of the LMP2Avax vaccine (Figure 4D).

## 4. Discussion

EBV, formally known as human gammaherpesvirus 4, is responsible for infectious mononucleosis, multiple premalignant conditions, and various EBV-driven cancers. These cancers include Burkitt Lymphoma, Hodgkin’s lymphoma, gastric cancer, nasopharyngeal carcinoma, HIV-associated oral hairy leukoplakia, and numerous other lymphoproliferative disorders. Additionally, EBV infection is associated with nonmalignant diseases and significant autoimmune disorders [55]. The worldwide burden of EBV-associated cancer is approximately 150,000 deaths per year, which represents almost 2% of all deaths from cancers. This burden continues to grow. EBV-associated gastric and nasopharyngeal carcinomas are each responsible for over 60,000 cancer deaths per year, and the incidence of the latter is increasing [56]. In light of this burden, additional approaches to EBV immunotherapy are important. 

Here, we engineered synthetic consensus DNA vaccines of modified EBV latent proteins to generate immune responses which could impact tumor regression. Latent proteins are present in both lymphomas and carcinomas associated with EBV, and these have been studied as potential targets in various immunotherapeutic strategies. Currently there is no licensed approach for EBV immunotherapy. Cellular therapies have been studied in small trials and have shown some important effects [57,58]. However, these were early studies and additional approaches would be highly beneficial. 

Along these lines, work in the HPV setting with SynCon DNA vaccines delivered by adaptive EP has evolved to be a robust approach for induction of antiviral cellular immunity, which can impact tumors and precancers in vivo [26,27]. We tested this approach here for a three-antigen synthetic DNA vaccine approach targeting the major EBV latent oncoproteins. We chose these antigen targets because they are present in EBV-associated cancers. Small trials of cellular therapies targeting EBNA1 [59] and LMPs [25,60,61] have shown improved outcomes against EBV-associated diseases. The high frequency of nasopharyngeal carcinoma concentrated in east Asia makes for a unique environment to test prophylactic and therapeutic approaches targeting the virus [62]. The frequency of Hodgkin’s lymphoma in Europe and its temporal association with infectious mononucleosis offers another opportunity [7]. The growing burden of EBV in the US suggests immunotherapy for nasopharyngeal and gastric cancer as well as association of EBV with more common autoimmune disorders may also be important to consider as amenable to robust immunotherapy approaches [61]. 

Synthetic DNA vaccines can drive in vivo immune responses via MHC class I and II presentation through their delivery of and intracellular production of genetically encoded antigens. Newer delivery approaches have resulted in the generation of more consistent and robust immunity that can target cancer in the clinic [26,27]. Here we show that these designed latent antigen vaccines elicit significant cytotoxic T lymphocyte responses against the encoded vaccine targets EBNA1vax and LMP2Avax, which showed dominant CD8 T cell responses in vivo. These cellular responses are important in protecting mouse models from EBV antigen-expressing tumors in murine vaccine models, as recently shown in a novel heterologous prime-boost approach that impacted an EBNA1 tumor challenge [63]. Importantly, LMP2Avax-induced immunity protected against tumor growth in a TC-1 challenge model where LMP2A was targeted by the immunization. The immune responses produced by EBNA1vax and LMP2Avax merit further study. In addition, continued engineering may be interesting in this regard, as DNA delivery of LMP1 as an immunogen can clearly impact tumor growth as a standalone antigen in some models [28]. Combination development for this group of immunogens appears worthy of additional attention. 

Recent developments in the DNA platform in formulation, engineering and delivery by adaptive EP have led to improved immune potency and improved consistency in clinical studies [26,27]. In these studies, we noted that the vaccines were biased towards driving highly desired CD8 immunity against the vaccine targets over CD4 immunity. This CD8 bias may be particularly relevant for clearing virally infected cells by cytotoxic T lymphocyte induction that would ultimately kill tumor cells. These latent antigen vaccines could be studied in the context of epithelial tumors, such as gastric and nasopharyngeal carcinomas, among others. The addition of checkpoint inhibitors in the context of these immunizations, as we have reported for HPV, might also be of interest for impacting EBV-related tumor progression [26,27,64].

## 5. Conclusions

There is a great need for new approaches targeting EBV, against which there are no licensed vaccines or immunotherapies available. Acute infection can lead to infectious mononucleosis, and the risk of autoimmune diseases such as multiple sclerosis is increased following symptomatic infection. Immunotherapy targeting conserved, expressed, and oncogenic viral genes has the potential to drive immunity that impacts EBV-associated cancers. Here we generated synthetic DNA immunogens targeting the EBV latent proteins EBNA1, LMP1, and LMP2. These engineered SynCon DNA vaccines were delivered by Cellectra EP into mice to study their immune responses. The combination of immunogens generated significant CD8 T cell responses. In addition, these responses impacted tumor growth in a mouse challenge model. Further study of this combination synthetic DNA approach in EBV-driven disease is warranted. 

## Figures and Tables

**Figure A1:**
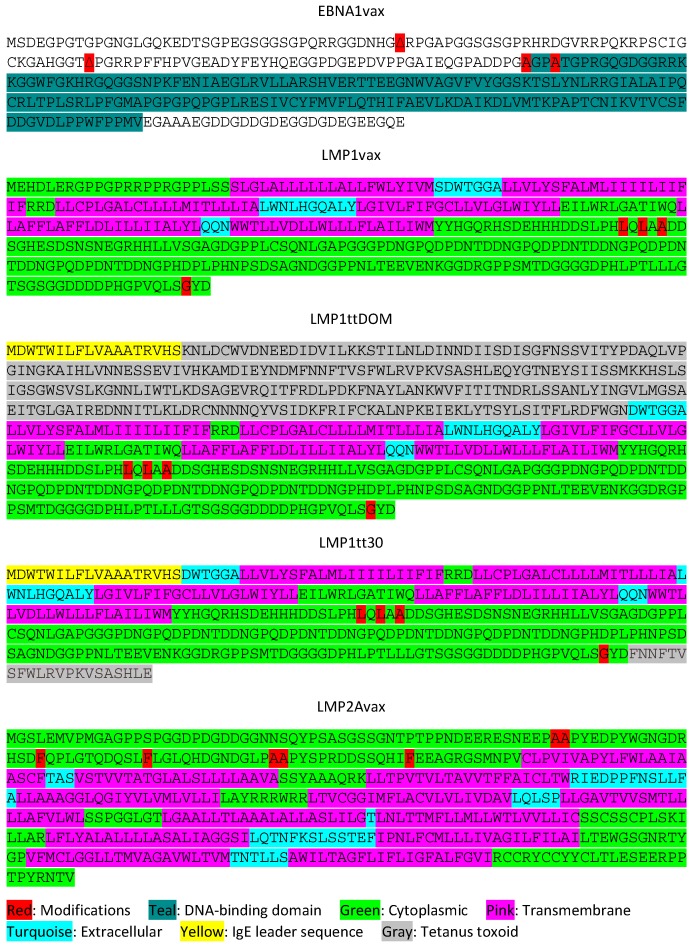
EBNA1vax contains deleted regions of glycine-arginine and glycine-alanine repeats marked by Δ (12 and 310 amino acid deletions) and has its DNA-binding domain highlighted. LMP1vax and LMP2Avax have their cytoplasmic, extracellular, and transmembrane regions indicated. Modifications to engineer LMP2Avax derivatives are labeled as well.

**Figure A2:**
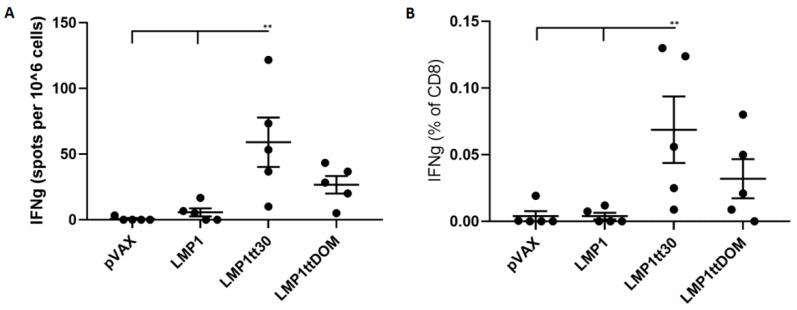
Engineered LMP1 vaccines enhance cellular immunity. The LMP1 antigen was truncated at the N-terminal, received an IgE leader sequence, and had tetanus toxoid added to its sequence. Splenocytes were stimulated with the 5 strongest MHC class I peptides to LMP1, as predicted in silico, after C57BL/6 mice were vaccinated. (**A**) IFNγ ELISPOT results showing an average of 59 sfu for LMP1tt30. (**B**) Flow cytometry data showing improved CD8 responses following plasmid engineering.
